# Hate the wait? How social inferences can cause customers who wait longer to buy more

**DOI:** 10.3389/fpsyg.2022.990671

**Published:** 2022-10-18

**Authors:** Nira Munichor, Alan D. J. Cooke

**Affiliations:** ^1^The Graduate School of Business Administration, Bar-Ilan University, Ramat-Gan, Israel; ^2^Department of Marketing, Warrington College of Business Administration, University of Florida, Gainesville, FL, United States

**Keywords:** waiting time, queues, social inferences, consumer behavior, purchases

## Abstract

Waiting is a mundane yet inevitable customer experience. Surprisingly, little research has analyzed the effects of waiting on subsequent customer behavior. The current research explores a counterintuitive effect of waiting times on behavior during a shopping trip: Longer waits, compared with shorter waits, can lead to a larger number of purchases despite generating more negative emotional reactions. Results of a field study and three lab experiments demonstrate this effect in the context of waiting for hedonic products. Consistent with a social-inference account, the experiments further show that the effect of waiting duration occurs when wait times are thought to depend on others’ preferences. This article explores the multifaceted effects of waiting duration on purchase behavior of hedonic products and sheds light on the social cognitions that underlie these effects.

## Introduction

Waiting is an abhorred yet inevitable customer experience, imposed by firms’ use of lines as a solution to limited firm resources and fluctuations in demand (Schwartz, 1975). For customers, time is a scarce resource, so waiting potentially carries economic costs, such as reduced productivity (Becker, 1965; Hui and Tse, 1996), and psychological costs including boredom, frustration, anxiety, and anger (Maister, 1985; Osuna, 1985; Carmon et al., 1995; Zakay, 2014). From the perspective of firms, these costs to the customer could potentially influence important factors such as customer patience, loyalty, and retention (Taylor, 1994; Evangelist et al., 2002; Janakiraman et al., 2011; Craig et al., 2017; De Vries et al., 2018). On the assumption that long waits lead customers to reduce investment in a product or firm (whether of time, money, or attention), practitioners have developed various ways to manage waiting, including through new technologies designed to shorten waiting times (e.g., Apple’s SmartLine app for managing restaurant bookings, Apple, 2015; John Lewis’s “click & collect,” Anderson, 2015; or hotel check-in apps, Sathyanarayanan, 2014). However, contrary to this common intuition, we suggest there exist specific conditions under which customers may actually decide to purchase and consume more after a longer rather than a shorter wait.

Although queues and waiting have been a part of the research agenda in various domains (e.g., Hui et al., 1998; Zohar et al., 2002; Gorn et al., 2004; Munichor and Rafaeli, 2007; Kumar and Krishnamurthy, 2008), their impact on customer actual behavior has received little research. Especially lacking are studies examining how waiting affects purchasing behavior. The current research bridges this gap by investigating the impact of wait durations on subsequent (hedonic) purchase and consumption decisions. We show that although customers experience longer waits as more negative than shorter waits (in keeping with previous research), wait durations can have a positive impact on subsequent purchases and consumption. We propose that this effect arises when a long wait is interpreted as a sign that the good or service is worth waiting for because the item is assumed to be valued by others based on a social-inference process.

Thus, our goals in this paper are twofold: First, we explore the nuanced and counterintuitive relationship between waiting and customers’ subsequent purchase behavior. We demonstrate that despite greater annoyance from longer waits, they can have a positive impact on subsequent purchases and consumption (of hedonic products). This result repudiates the common business doctrine that making customers wait is harmful and can only damage the firm’s bottom line. Second, we test one of the possible psychological explanations for longer waits leading to more consumption—social inference. We propose that customers infer that the length of a wait signals the popularity or quality of the products being purchased. When waits are long and customers are able to make this inference, they will tend to purchase more of the products, but when waits are attributed to other factors, they will not. We demonstrate the relationship between wait duration and purchasing behavior in one field study and three lab experiments. The lab experiments systematically manipulate the putative explanation for participants’ purchase and consumption decisions following a wait, directly demonstrating the moderating effect of social inference on the relationship between waiting and consumption.

## Conceptual background and hypotheses development

### The impact of waiting on customers

Aside from some exceptions (e.g., waiting for an aversive event [Miller et al., 2008] or anticipating one-of-a-kind experiences, such as a kiss from a movie star [Loewenstein, 1987]), queueing—or waiting for service—is known to elicit negative emotional reactions, from boredom to anxiety and distress (Osuna, 1985; Dubé et al., 1991; Zhou and Soman, 2003; Zakay, 2014). Unsurprisingly, then, many studies have found that customers evaluate the service received after a wait as less satisfactory (Hui et al., 1998; Dellaert and Kahn, 1999; Whiting and Donthu, 2006) and express lower future patronage intentions (Baker et al., 2002; Craig et al., 2017). Yet despite abundant literature documenting a wide range of (negative) reactions to queue-waiting, direct measures of behavior in waiting studies are rare. Those that exist focus mainly on customer patience, usually measured by the amount of time customers wait before abandoning a line (Zhou and Soman, 2003; Munichor and Rafaeli, 2007; Janakiraman et al., 2011). Few studies have examined the impact of waiting on purchase behavior—a surprising lacuna, given that such work could greatly enrich our knowledge of the effects of waiting.

In one exception, Koo and Fishbach (2010) showed that longer lines behind a customer increase the amount of money spent upon reaching the front of the line. Thus, some aspects of queues may actually positively affect firms’ bottom lines. This is in keeping with findings that a long line behind the customer improves customers’ affective state and reduces rates of abandoning (Zhou and Soman, 2003). We propose that customers may spend more upon reaching the head of the line *after a prolonged wait*, which increases customers’ emotional distress and has been connected to negative customer reactions (Clemmer and Schneider, 1993; Taylor, 1994; Antonides et al., 2002).

### Wait duration

The duration of a wait is assumed to be key to customer reactions (Brady and Cronin Jr., 2001). Time may be even more dominant in the contemporary experience of waiting, when a growing number of lines are virtual, and customers cannot see those queueing with them. Theoretical works (Osuna, 1985), as well as empirical studies (Clemmer and Schneider, 1993; Taylor, 1994; Antonides et al., 2002; Baker et al., 2002; Whiting and Donthu, 2006; Kumar and Dada, 2021), have connected prolonged waits (both objective and subjective) with negative customer reactions. Yet other studies have failed to find similar relationships, whether with respect to subjective (Hui and Tse, 1996; Hui and Zhou, 1996; Hui et al., 1997; Cameron et al., 2003; Munichor and Rafaeli, 2007) or objective (Selvidge et al., 2002; Rose et al., 2005) wait times. There is also evidence that different aspects of a queuing experience can affect customers’ reactions beyond either the objective passage of time or the customer’s subjective experience of it. For instance, Ivanic (2015) found that customers who were told that they had earned high status in a loyalty program preferred a queue framed as exclusive to a nonexclusive queue, even though the former involved a longer wait, to reinforce their status. These findings hint at complex, and perhaps unintuitive, effects of wait duration on customers.

One possible reason for the complex effects of wait duration may be the coexistence of negative and positive implications in longer waits. A customer’s behavior following a wait of a certain duration can be seen as resulting from a “mindsponge process,” in which information existing in mind is incorporated with new information about negative and positive implications of the wait to make decisions that maximize perceived benefits and minimize perceived costs (Vuong and Napier, 2015; Vuong, 2022). On the negative (i.e., costs) side, longer waits imply a greater loss of time, a scarce resource that could be utilized for the pursuit of other goals, with all the frustration and annoyance that this implies (Leclerc et al., 1995; Hui and Tse, 1996). At the same time, cognitions about the nature of and reasons for the prolonged wait may affect customers’ beliefs about the benefits of the good or service they are waiting for, and thus their decision regarding how much to buy and consume once they reach the point-of-purchase. Specifically, longer waits may serve as a positive signal of greater benefits in the waited-for good or service. For example, customers may perceive longer waits as an indication of higher demand for the product, which suggests that the product has a higher value, and consequently buys more (as elaborated below). Indeed, Giebelhausen et al. (2011) found that customers who waited for services and products perceived them as being of higher quality than customers who did not wait.

Given the coexistence of negative and positive implications, we suggest that in some cases, a longer wait may lead to *increased* purchases and consumption even when the prolonged wait results in more negative emotional states:

*Hypothesis 1a:* Customers may purchase and consume more of the waited-for item after a longer (vs. shorter) wait.

*Hypothesis 1b:* A longer (vs. shorter) wait increases customers’ annoyance.

We suggest greater annoyance and increased consumption may cooccur when wait duration serves as a value signal of other customers’ preferences. It should be noted that other factors could also potentially contribute to increasing purchases and consumption following longer waits. For example, a customer’s decision to continue waiting despite a long wait may signal one’s own preferences (Koo and Fishbach, 2010). Longer waits may also impose higher demands on customers than shorter waits, hindering customers’ attempts to exert self-control (Muraven and Baumeister, 2000; Baumeister, 2002; Vohs and Faber, 2007). In addition, longer waits may encourage customers to buy and consume more in order to repair negative feelings (Arnold and Reynolds, 2009) or to compensate for the perceived cost of waiting (Arkes and Blumer, 1985; Thaler, 1985; Ülkü et al., 2020). While recognizing that the relationship between waiting and purchase decisions is likely multiply-determined, we focus our investigation on social inferences that customers may draw from their waiting duration.

### Social inferences from wait duration

Customers’ judgments, decisions, and behaviors are known to be influenced by the behavior of others (Bearden and Etzel, 1982; Hellofs and Jacobson, 1999; Colm et al., 2017). Specifically, customers observe the behavior—and, in particular, the choices—of others in order to draw inferences by which to make informed decisions, particularly in cases where they have only incomplete information (Deutsch and Gerard, 1955; Burnkrant and Cousineau, 1975; Colm et al., 2017). For example, a descriptive norm, namely the perception that most people make the same choice, may be interpreted as an indication of what will likely be an effective and adaptive action (Cialdini et al., 1991). Hence, as long as value is not driven by product exclusivity, high demand for a particular product may lead customers to infer that the product is of high quality and value (Hellofs and Jacobson, 1999; van Herpen et al., 2009; Steinhart et al., 2014; Goedegebure et al., 2021). Along these lines, willingness to be vaccinated is dependent on the number of others vaccinated (Hershey et al., 1994), purchasing of digital books is enhanced with a larger number of consumption-clicks by previous site users (Ding and Li, 2019), and online bidders irrationally choose auctions with more existing bids (Simonsohn and Ariely, 2008). More closely related to our research, longer lines at restaurants can attract more customers, especially in tourist areas, where customers are uncertain about the quality of the restaurant (Raz and Ert, 2008).

When a physical line is absent, and customers cannot see how many others are waiting ahead of or behind them, the time spent waiting often serves as a general proxy for queue length (Whitt, 1999). Therefore, customers may use waiting time as an indication of value in the same way they use queue length. That is, customers may assume that if a good or service entails a longer wait, it is more popular, and, in turn, more valuable. The attribution of greater value may then lead customers to buy more. More formally, assuming that a longer wait serves as a basis for social inference-making about the item’s value, we predict that:

*Hypothesis 2:* A longer (vs. shorter) wait will lead to subsequent increased purchases and consumption when wait times can be considered indicative of other customers’ desire for the waited-for item.

## Materials and methods

We tested our predictions in four studies revolving around purchases and consumption of hedonic products. We received IRB approval for the procedures. Study 1 tests the basic proposed effect of wait duration on subsequent purchasing and consumption behavior in the field. Study 2 replicates this effect in the lab and shows that longer waits lead to more purchases and increased consumption despite eliciting more negative emotional reactions. Studies 3a and 3b provide support for our proposed social-inference mechanism by showing that a long (vs. short) wait only increases the number of items participants choose to consume after waiting (Study 3a) and the number of products participants purchase (Study 3b) when they believe their waiting time reflects other participants’ preferences.

### Study 1: Wait duration and expenditures at a Café

The purpose of Study 1 was to demonstrate in a real consumption environment that customers who wait longer in line may purchase more. We conducted the study in a café where customers could select and purchase items only at the cashier stand. We documented the length of time customers waited to test whether durations were positively related to the sum of money customers spent on items after waiting.

#### Participants

Two hundred and thirty-four customers were observed in the study. Seventeen of these customers (7%) did not need to wait for service, and critical variables of 11 other customers were not fully documented. All these customers were omitted from further analyses, yielding a valid sample size of 206 customers (57% female).

#### Procedure

Unobtrusive observers sampled café patrons at a variety of hours over four different days. They documented the time of day when the transaction took place (as a proxy for demand fluctuations), customers’ line-entrance and service-entrance times, the number of people ahead at line-entrance, the number of people behind at service-entrance, and the number of people who accompanied the paying customer to the café (as a proxy for the number of people for whom the focal customer paid). Cashier data supplied the amount of money each customer spent on items purchased at the cashier stand.

#### Results

No customers abandoned the line before reaching the cashier stand. The length of each customer’s wait was calculated as the difference between that customer’s line-entrance and service-entrance times. Table 1 provides descriptive statistics and tests of normality for the variables in the dataset.

**Table 1:** Descriptive statistics and tests for normality of key variables in Study 1.

Variable	Mean	Standard deviation	Minimum	Maximum	Kolmogorov–Smirnov’s *D*	Skewness
Wait duration (min.)	1:29	1:14	0:03	9:01	0.14^***^	2.36
Expenditures ($)	7.44	0.34	1.80	31.36	0.13^***^	1.86
Number of people ahead at line-entrance	2.74	2.26	0	11	0.19^***^	1.39
Number of people behind at service-entrance	1.83	2.18	0	10	0.23^***^	1.38
Number of companions	0.33	0.61	0	4	0.42^***^	2.43

*** *p* < 0.001 (deviation from normal distribution).

As summarized in Tables 2A,B, and in line with Hypothesis 1a, a regression showed that wait durations were positively related to expenditures [*t*(204) = 2.64, *b* = 0.05, *p* = 0.009, *R*^2^ = 0.06]. A regression of the log-transformed wait durations on the log-transformed expenditures (to correct for the fact that neither variable was distributed normally; *t*(204) = 2.55, *b* = 0.124, *p* = 0.01, *R*^2^ = 0.03) and a regression that focused only on customers who arrived alone at the café [*t*(145) = 2.93, *b* = 0.062, *p* = 0.004, *R*^2^ = 0.06] also yielded similar results.

**Table 2A:** Summary of analyses of the relationships between wait durations and expenditures in Study 1.

Variable	Model 1 (no control variables)	Model 2 (no control variables; customers who arrived alone)	Model 3 (with control variables)
Wait duration	2.64^**^	2.93^**^	1.86^+^
Time of day	–	–	1.58
Number of people behind at service-entrance	–	–	0.76
Number of companions	–	–	2.98^**^

Entries in the table represent *t*-statistics.

+ *p* < 0.1 and

** *p* < 0.01.

**Table 2B:** Summary of analyses of the relationships between log-transformed wait durations and log-transformed expenditures in Study 1.

Variable	Model 1 (no control variables)	Model 2 (no control variables; customers who arrived alone)	Model 3 (with control variables)	Model 4 (with log-transformed control variables)
Log-transformed wait duration	2.55^**^	2.09^*^	1.91^+^	1.86^+^
Time of day	–	–	1.40	1.42
Number of people behind at service-entrance	–	–	0.62	–
Log-transformed number of people behind	–	–	–	0.82
Number of companions	–	–	2.44^*^	–
Log-transformed number of companions	–	–	–	2.13^*^

Entries in the table represent *t*-statistics.

+ *p* < 0.1,

* *p* < 0.05, and

** *p* < 0.01.

The positive relationship between wait durations and expenditures remained (marginally) significant [*t*(199) = 1.86, *p* = 0.06] in a model [*F*(4,199) = 4.92, *p* < 0.001, *R*^2^ = 0.09] that controlled for the time of day when the transaction took place [*t*(199) = 1.58, *p* > 0.1], number of companions [*t*(199) = 2.98, *p* = 0.003], and number of people behind the customer at the end of their wait [*t*(199) = 0.76, *p* > 0.4]. Likewise, the positive relationship between log-transformed wait durations and log-transformed expenditures was (marginally) significant [*t*(199) = 1.91, *p* = 0.06] in a model [*F*(4,199) = 3.79, *p* = 0.005, *R*^2^ = 0.07] that controlled for the time of day when the transaction took place [*t*(199) = 1.40, *p* > 0.1], number of companions [*t*(199) = 2.44, *p* = 0.015], and number of people behind the customer at the end of their wait [*t*(199) = 0.62, *p* > 0.5]. The relationship between log-transformed wait durations and log-transformed expenditures remained (marginally) significant [*t*(199) = 1.86, *p* = 0.06] also in a model [*F*(4,199) = 3.50, *p* = 0.009, *R*^2^ = 0.07] that controlled for the time of day [*t*(199) = 1.42, *p* > 0.1], log-transformed number of companions [*t*(199) = 2.13, *p* = 0.03] and log-transformed number of people behind [*t*(199) = 0.82, *p* > 0.4].

#### Discussion

The results of this field study show that positive relationships between how long customers wait and how much they spend do exist in realistic service settings. Of course, the observational nature of this field study does not allow for causal inferences regarding the nature of this relationship. Furthermore, there are many prosaic ways such a positive relationship might emerge in Study 1 that are unrelated to customers’ social inferences based on the wait (e.g., longer waits may serve as a self-preference signal, hinder self-control attempts, or encourage compensation). Thus, we turned to laboratory experiments to better explore this phenomenon, and the role played in it by social inference-making.

### Study 2: The effect of wait duration on the number of items selected

Study 2 aimed to provide direct support for our hypothesis that customers may purchase and consume more after longer waits by demonstrating this effect in a controlled laboratory environment. We used a computer-based task that mimicked waits in many typical service environments (e.g., counter-service restaurants) using decisions that had real consequences for the participants. During the task, participants waited while videos were purportedly downloaded to their computer. At the end of their wait, participants selected how many videos they wanted to watch from a list. The number of videos chosen was our critical variable. We hypothesized that following a longer wait, as compared with a shorter wait, participants would choose to consume more videos.

Study 2 also allowed us to measure emotional reactions to the wait, and to test whether longer waits increase customers’ annoyance. This further served as an external validity test of our waiting task: if the task reliably mimicked typical waits, the longer wait should produce more negative emotional reactions than the shorter wait. The procedure thus enabled us to rule out the possibility that our longer wait inadvertently induced positive emotional reactions compared with our shorter wait, and that these reactions could explain any increase in consumption. Finally, in this study, we asked participants to evaluate their consumption as part of the cover story, thus incurring personal consumption costs that might not exist in most service environments. To test whether the effect of the time invested in waiting, being a cost customers pay in order to consume the item being waited for (Beran, 2015), is contingent upon the costs involved in that consumption, we also manipulated the consumption cost.

#### Participants

Eighty-two undergraduate students at a USA university (44% female, *M*_age_ = 21.0) who listed themselves on the lab’s recruitment ad in advance and showed up at the designated time participated in this study for course credit. Participants were randomly assigned to a 2 (wait duration: short vs. long) × 2 (consumption cost: low vs. high) between-subjects design.

#### Procedure

All participants were invited for a fixed session time of 40 min in the university lab, after which they all received the same course credit as a fee for their participation. We told them that we were interested in factors that characterize effective speakers. We also told participants that they would be given a list of video clips to choose from and that their task would be to view the chosen clips and evaluate the speakers’ ability to convey their message along several dimensions. Participants were told that they could choose as many or as few clips as they wished, but that upon completing the evaluation task, they would be given other tasks to fill the remainder of the session. This was done to eliminate any extrinsic motivation to select fewer videos. We also informed participants that depending on the system’s load, they might need to wait for the media files to download.

After participants read the instructions, they were given a demonstration of the task to be performed, so they were exposed to the cost involved in evaluating each video clip before they made any choice. We manipulated the consumption cost by using two sets of evaluation items. Participants in the low-cost condition were asked to evaluate each presenter along four 7-point Likert-scale dimensions, while participants in the high-cost condition were presented with ten different dimensions, some of which comprised open-ended questions.

Then, we asked participants to choose one of two video types: either stand-up comedy or lectures. This choice allowed waiting times to serve as a basis for inference-making, providing participants with a decision that could be expected to affect their wait time (stand-up comedy could be assumed to be more popular, increasing the load on the system). However, we expected that most participants would gravitate to stand-up comedy, which would enable us to focus our analyses on one product type (thereby removing a confounding factor, namely the differences between video types in the distributions of the number of clips chosen, from the analysis).

The next screen prompted participants to wait while the media files were downloaded. In reality, we manipulated the wait duration, which was 2:06 min in the short-wait condition and 6:06 min in the long-wait condition. Participants were instructed to stay focused on the screen during their wait to ensure that the waiting duration was fully perceived.

**Figure 1:**
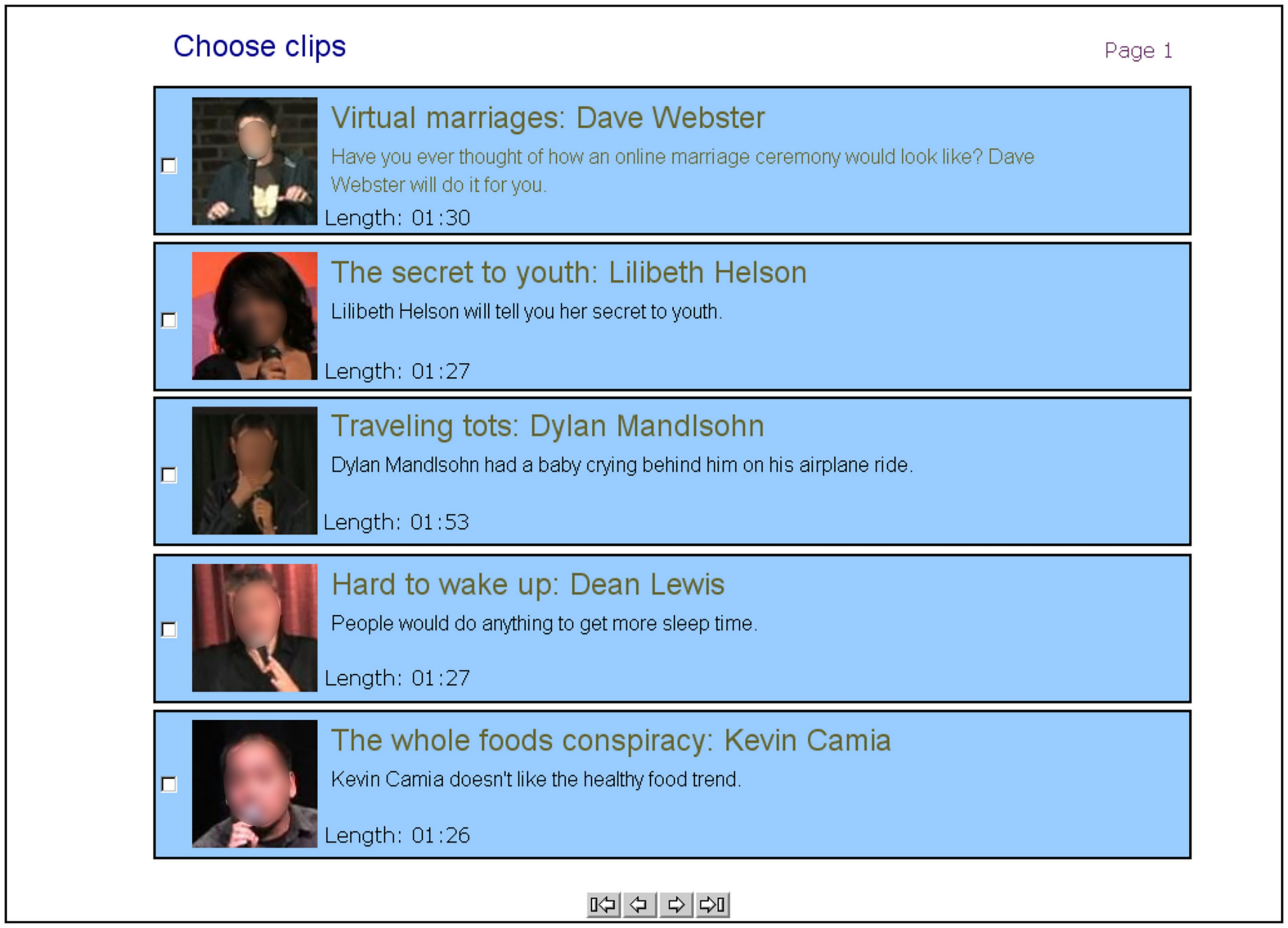
A screenshot of one of three pages presenting a clip list in Studies 2 and 3a. Participants could navigate between pages using the buttons at the bottom of the screen. Participants were instructed to choose as many or as few of the presentation clips as they want, as the number of clips chosen is the critical variable (DV) in the experiments.

After the wait, participants received a list of 12 videos. As Figure 1 presents, each video description contained the video title, the presenter’s name and photo, a one-sentence description, and the length of the clip (1:19–1:53 min). Participants were next asked to select the clips they wanted to view. They then watched the chosen videos (note that the specific choices each participant made shaped their consumption experience, creating an individual self-determined experience as in real-life). Participants evaluated each chosen video, using the same set of evaluation items as in the demonstration. At the end of the task, participants indicated whether they had needed to wait for the files to download (yes/no; a question designed to disguise our manipulations). Those who indicated a wait also reported the duration of their wait (an open-ended question) and rated their level of annoyance with the wait on a 7-point scale (1 = “not at all” and 7 = “very much”). Participants also were asked to describe the reasons for their choice of presentation type and clip selection (open-ended questions). No one mentioned a wait-related reason for either of these choices. Finally, participants completed several filler questions and demographic measures (gender, age, and native language). At this point, participants were debriefed before being given a packet of unrelated paper-and-pencil questionnaires to complete until the session was over.

#### Results

As expected, most participants chose to watch stand-up comedy: 75 of the 82 participants (91.5%). Thus, in our analyses, we could hold product type (and the associated distribution) fixed and focus on the 75 participants who chose to watch comedy clips.

The number of comedy clips participants selected, our main critical variable, ranged from 1 to 12 (*M* = 5.05, SD = 2.69). As summarized in Tables 3, a 2 (wait duration: short vs. long) × 2 (consumption cost: low vs. high) ANOVA on the number of clips selected revealed a significant main effect of wait duration [*F*(1,71) = 5.39, *p* = 0.02, *η*^2^ = 0.071]. In line with Hypothesis 1a, participants chose to watch more clips when the wait was long (*M* = 5.77, SD = 2.76) than when the wait was short (*M* = 4.42, SD = 2.49). Consumption cost had a marginally significant effect on the number of clips [*F*(1,71) = 3.36, *p* = 0.07]: Participants selected slightly more clips in the low-cost condition (*M* = 5.59, SD = 2.84) than in the high-cost condition (*M* = 4.47, SD = 2.41). The effect of the interaction between wait duration and consumption cost was statistically insignificant [*F*(1,71) = 1.09, *p* = 0.30].

**Table 3:** Summary of analyses of the effects of wait duration and consumption cost on the number of comedy clips selected for watching, perceived wait time, and wait annoyance in Study 2.

Variable	Number of clips	Log-transformed number of clips	Perceived wait time	Wait annoyance
Wait duration	5.39^**^	5.00^**^	3452.74^***^	9.47^**^
Consumption cost	3.36^+^	2.69	0.08	0.36
Wait duration × consumption cost	1.09	0.16	0.46	0.78

Entries in the table represent *F*-statistics.

+ *p* < 0.1,

** *p* < 0.01, and

*** *p* < 0.001.

A KS test for normality showed that the distribution of the number of clips participants selected deviated significantly from normal (*D* = 0.15, *p* < 0.001, skewness = 0.99, SE = 0.28). We therefore also analyzed the log transformation of this measure. A 2 (wait duration: short vs. long) × 2 (consumption cost: low vs. high) ANOVA on the log-transformed number of clips selected again revealed a significant main effect of wait duration [*F*(1,71) = 5.00, *p* = 0.03, *η*^2^ = 0.066]. Neither the main effect of consumption cost nor the effect of the interaction between duration and cost on the log-transformed number of chosen clips was statistically significant (all *p*s > 0.1).

Two participants (one in the short-wait condition and one in the long-wait condition) reported not waiting, and therefore did not provide answers to the perceived wait time and perceived annoyance questions. Due to a technical problem in one of our computers, the answers of two other participants (in the long-wait condition) were not recorded. All four of these participants were dropped from the subsequent analyses. A comparison of the reported wait times confirmed that participants were aware of their wait time (*M*_short_ = 124.97 s., SD_short_ = 16.18 vs. *M*_long_ = 364.47 s., SD_long_ = 17.59, *F*(1,67) = 3452.74, *p* < 0.001, *η*^2^ = 0.98). Neither the main effect of consumption cost nor the effect of the interaction between duration and cost on reported wait times was statistically significant (both *ps* > 0.4).

In line with Hypothesis 1b, wait annoyance was significantly affected by wait duration [*F*(1,67) = 9.475, *p* = 0.003, *η*^2^ = 0.12]. Participants perceived the long wait to be more annoying than the short wait (*M*_long_ = 6.22, SD_long_ = 1.29 vs. *M*_short_ *=* 4.95, SD_short_ = 2.02). Neither the main effect of consumption cost nor the effect of the interaction between duration and cost on perceived wait annoyance was significant (both *p*s > 0.3).

#### Discussion

Study 2 provides causal evidence for the effect of wait duration on subsequent purchases. In what might seem counterintuitive, longer waits, despite their negative emotional impact, led participants to select more items for consumption than shorter waits. In addition, we found no evidence supporting the possibility that consumption costs (i.e., resources invested in consumption evaluation) alter the effect of wait duration on the amount selected following a wait. This may be because consumption costs, as opposed to the effort invested in waiting, are experienced only after the purchase decision has been made, and therefore are subject to different inference-making processes. Study 2 therefore also hints at the role of social inferences in the effect of wait duration on item selection, which we explore more systematically next.

### Studies 3a and 3b: Social inferences from the wait duration

Studies 3a and 3b examined whether the function of wait duration as a signal of other customers’ preferences plays a role in the effect of wait duration on subsequent purchase and consumption decisions. We posit that as wait times lengthen, customers infer that the item being waited for is more popular and therefore more valuable. If so, longer (vs. shorter) waits should increase subsequent purchases and consumption only when the wait duration is perceived as indicative of other customers’ behavior (i.e., indicative of a descriptive norm of purchasing and consuming). Thus, to test whether social inferences can account for the effect of wait duration on subsequent purchasing and consumption behavior, Studies 3a and 3b manipulated both wait duration and whether the wait duration could be interpreted as evidence of others’ interest in the items being waited for.

#### Study 3a: Video clip selection

Study 3a served as an initial test of the social-inference process account. To test the hypothesis that longer (vs. shorter) waits increase purchases when they serve as a signal that other customers value the good(s) on offer, we used a procedure similar to that used in Study 2, but ascribed wait times either to other participants’ preferences or to unrelated system load. If longer waits increase subsequent purchases in the former case but not the latter, Study 3a would provide evidence supporting the social-inference account.

##### Participants

Ninety-five undergraduate students at a United States university (56% female, *M*_age_ = 20.2) who listed themselves on the lab’s recruitment ad in advance and showed up at the designated time participated in this study for course credit. Participants were randomly assigned to a 2 (wait duration: short vs. long) × 2 (signal about others’ behavior: present vs. absent) between-subjects design.

##### Procedure

The procedure that was carried out in the university lab was the same as in Study 2 with three exceptions. First, all participants watched stand-up comedy clips. Second, all participants evaluated each clip on the same 7-point scale. Finally, we told participants that they might have to wait for the clips to load, either because “the software can only process a few people at a time, and you may need to wait while other participants are being processed” (signal condition) or because “the server we use to host the clips is used for many other tasks in the college unrelated to the lab, and can be slow” (no-signal condition).

##### Results

**Figure 2:**
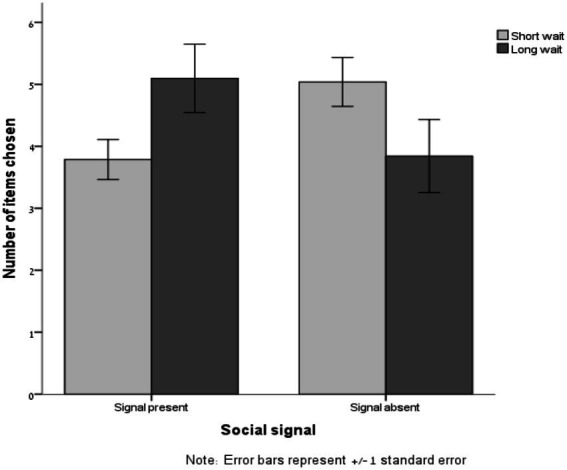
Number of clips selected for consumption as a function of wait duration (short vs. long) and availability of social signal (present vs. absent; Study 3a).

The number of clips selected ranged from 1 to 10 (*M* = 4.44, SD = 2.24). As summarized in Tables 4, A 2 (wait duration: short vs. long) × 2 (signal about others’ behavior: present vs. absent) ANOVA on the number of clips selected revealed only an interactive effect of wait duration and signal [*F*(1, 91) = 7.60, *p* = 0.007, *η*^2^ = 0.077; all other *p*s > 0.9; see Figure 2]. In line with Hypothesis 2, when participants were told that their wait time was dependent on other participants’ choices, they chose to watch more clips when the wait was long (*M* = 5.10, SD = 2.53) than when the wait was short [*M* = 3.79, SD = 1.71; *F*(1,91) = 4.31, *p* = 0.04]. However, this pattern reversed when participants were told their wait time was dependent on unrelated system load, with participants choosing directionally fewer clips following the long wait (*M* = 3.84, SD = 2.57) compared with the short wait [*M* = 5.04, SD = 2.05; *F*(1,91) = 3.34, *p* = 0.07].

**Table 4:** Summary of analyses of the effects of wait duration and signal about others’ behavior on the number of comedy clips selected for watching, perceived wait time, and wait annoyance in Study 3a.

Variable	Number of clips	Log-transformed number of clips	Perceived wait time	Wait annoyance
Wait duration	0.02	0.38	1004.34^***^	10.96^**^
Signal about others’ behavior	0.00	0.05	7.47^*^	0.33
Wait duration × signal about others’ behavior	7.60^**^	7.67^**^	4.48^*^	2.72

Entries in the table represent *F*-statistics.

* *p* < 0.05,

** *p* < 0.01, and

*** *p* < 0.001.

A KS test showed that the distribution of the number of clips selected deviated significantly from normal (*D* = 0.13, *p* < 0.001, skewness = 0.42, SE = 0.25). We therefore also analyzed the log transformation of this measure. A 2 (wait duration: short vs. long) × 2 (signal about others’ behavior: present vs. absent) ANOVA on the log-transformed number of clips selected again revealed only an interactive effect of wait duration and signal [*F*(1, 91) = 7.67, *p* = 0.007, *η*^2^ = 0.078; all other *p*s > 0.5].

Three participants (two in the short-wait condition and one in the long-wait condition) reported not waiting, and therefore did not provide answers to the perceived wait time and perceived wait annoyance questions. Due to a technical problem, answers of another participant (in the long-wait condition) were not recorded. These four participants were dropped from the subsequent analyses. A comparison of participants’ reported wait times following the short and long waits confirmed that participants were aware of their wait times (*M*_short_ = 130.53 s., SD_short_ = 30.39 vs. *M*_long_ = 371.32 s., SD_long_ = 45.06, *F*(1,87) = 1004.34, *p* < 0.001, *η*^2^ = 0.92). Estimated wait times were also affected by the presence versus absence of a signal [*F*(1,87) = 7.47, *p* = 0.08; such that participants perceived a longer wait when wait time served as a signal], and by the interaction between wait duration and presence versus absence of a signal [*F*(1,87) = 4.48, *p* = 0.04]. However, participants estimated the long wait as longer than the short wait in both the signal [*M*_short_ = 132.81 s, SD_short_ = 37.85 vs. *M*_long_ = 388.70 s, SD_long_ = 37.96, *F*(1,87) = 594.40, *p* < 0.001] and no-signal conditions [*M*_short_ = 128.15 s, SD_short_ = 20.49 vs. *M*_long_ = 352.00 s, SD_long_ = 45.36, *F*(1,87) = 421.10, *p* < 0.001].

Once again, wait annoyance was affected by wait duration [*F*(1,87) = 10.96, *p* = 0.001, *η*^2^ = 0.11]. Participants perceived the long wait as more annoying than the short wait (*M*_long_ = 6.11, SD_long_ = 1.33 vs. *M*_short_ *=* 5.11, SD_short_ = 1.51). Neither the main effect of signal (present vs. absent) nor the effect of the interaction between duration and signal was significant (*p*s > 0.1).

##### Discussion

The results of Study 3a offer initial support for social inference-making as a mechanism underlying the effect of wait duration on subsequent purchase and consumption. A long (vs. short) wait led participants to select a greater number of items for consumption only when wait times were believed to be indicative of others’ preferences. When wait times were perceived to be independent of others’ choices, however, longer waits led to a slight reduction in the number of items selected. This pattern of results provides support for our theory that wait duration serves as an indication of other customers’ preferences, such that longer (vs. shorter) waits yield increased consumption when they are perceived to reflect greater product popularity and (therefore) value. This pattern also suggests that when a prolonged wait cannot be taken as a social signal, consumption may be determined by customers’ negative emotional reaction to waiting. The next study further explores the underlying mechanism behind social inference-making using a computer-based task that carried monetary consequences for participants.

#### Study 3b: Purchases

Study 3b aimed to provide additional evidence for our proposed social-inference account. In this study, we employed a task that mimicked waits in many typical service environments, using decisions with monetary consequences and products of varying utility for participants. This procedure allowed us to examine in as realistic a setting as possible whether longer (vs. shorter) waits cause customers to subsequently select and purchase more items for consumption when they believe their wait time is indicative of other customers’ preferences, but not when they believe their wait time is independent of others’ choices.

##### Participants

One hundred and twenty undergraduate students at a United States university (61.7% female, *M*_age_ = 19.9 years) who listed themselves on the lab’s recruitment ad in advance and showed up at the designated time participated in this study for course credit. Participants were randomly assigned to a 2 (wait duration: short vs. long) × 2 (signal about others’ behavior: present vs. absent) between-subjects design.

##### Procedure

Participants were invited for a session that included several experiments in the university lab. We gave them $7.50 as payment for completing an unrelated prior task and then asked them to participate in a study examining how they shop and make product choices. In the study, we first briefly described two online stores, one selling snacks and the other school supplies, and asked participants to choose one of them. We expected that most participants would gravitate to snacks, enabling us to focus our analyses on one product type. After participants made their choice, we told them they might have to wait for the store page to load, and that their wait would reflect either processing of other participants (signal condition) or unrelated system load (no-signal condition). Participants then had to wait for either 16 s (short-wait) or 5 min and 6 s (long-wait).

**Figure 3:**
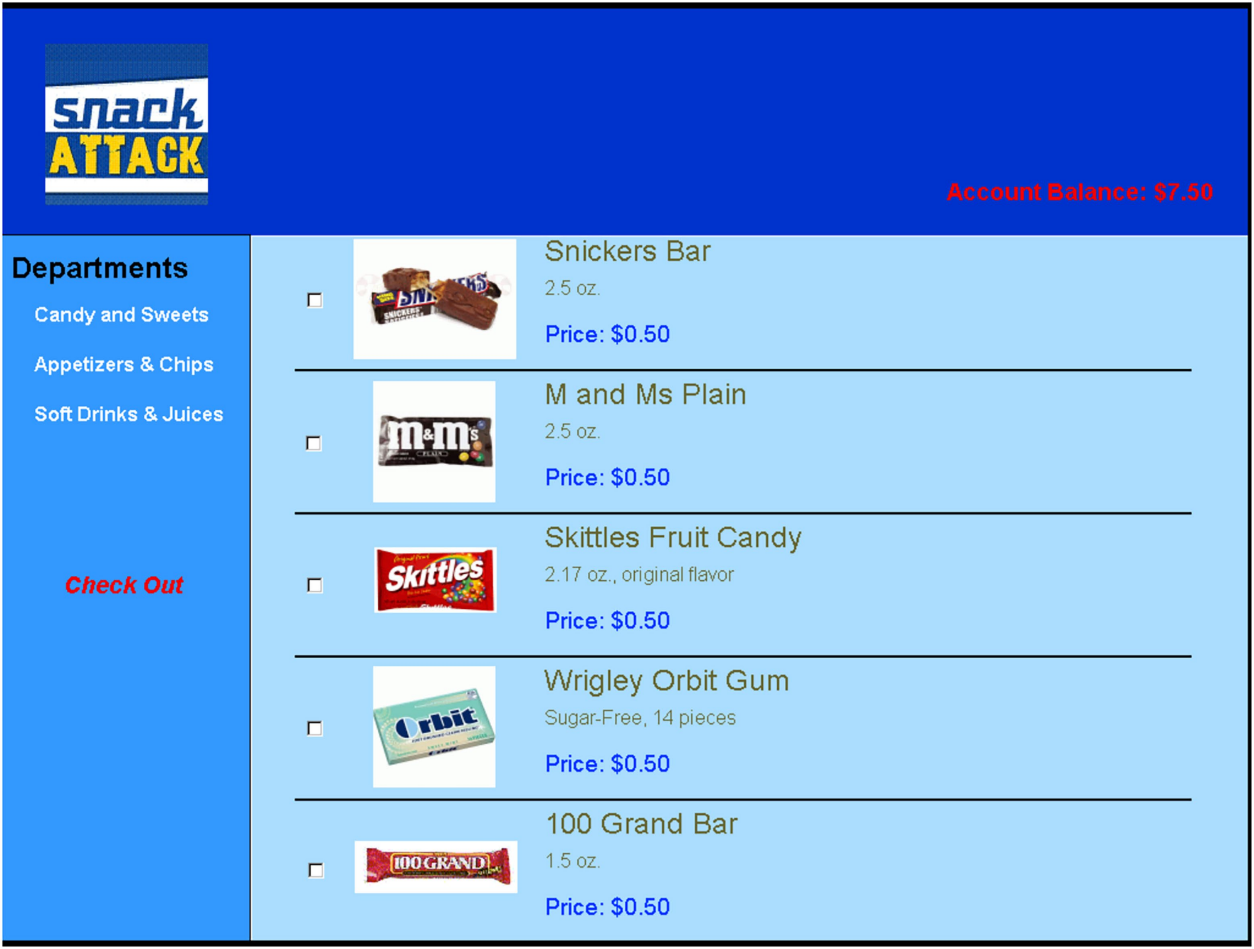
A screenshot of one of three pages presenting a snack list in Study 3b. Participants could navigate between pages using the menu at the left-hand side of the screen. Participants were instructed to choose as many or as few of the snacks as they want, as the number of snacks chosen is the critical variable (DV) in the experiment.

Once the wait was over, participants selected products to purchase from a list of 15 snacks or six school supplies, depending on their store selection (see Figure 3). The products were listed by name and accompanied by a photo and a brief description, along with their price (which was always 50 cents, notably less than the typical price for these products). We instructed participants to purchase as many of the products as they wanted.

After the task, participants reported how long the wait seemed to them on a 7-point scale (1 = “very short” and 7 = “very long”) and rated their annoyance with the wait (1 = “not at all” and 7 = “very much”). Participants also described the reasons for their store choice and product selection (open-ended questions). Finally, participants completed a few questions about their choices, as well as some filler questions and demographic measures (gender, age, and native language). They were then debriefed and given the products they had chosen and the remainder of their participation fee, if any.

##### Results

As expected, most participants chose to purchase snacks: 86 participants of the 120 participants (71.7%). Thus, in our analyses, we could again hold product type fixed and focus on the 86 participants who chose to purchase snacks. Of the 86 participants in the snack group, two outlier participants purchased the maximum number of products (spending more than their participation fee, arguably due to the abnormally low prices), and three indicated during the debriefing that they had surmised we were studying the effect of waiting on their decisions. These five participants were deleted from the analysis.

**Figure 4:**
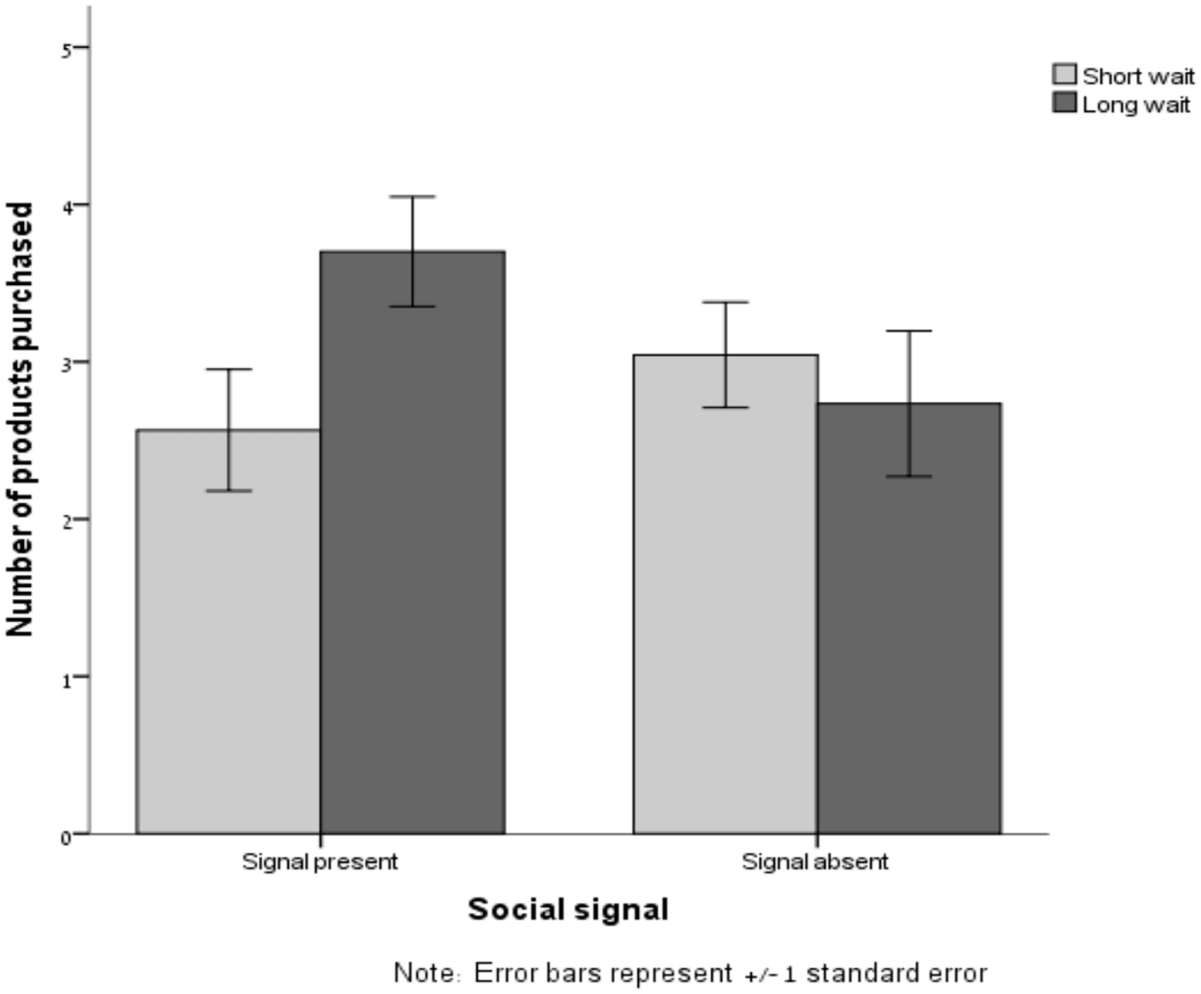
Number of snacks purchased as a function of wait duration (short vs. long) and availability of social signal (present vs. absent; Study 3b).

The number of snacks participants purchased ranged from 0 to 6 (*M* = 3.01, SD = 1.73). As summarized in Tables 5, a 2 (wait duration: short vs. long) × 2 (signal about others’ behavior: present vs. absent) ANOVA on the number of snacks purchased revealed a significant effect of wait duration [*F*(1, 77) = 57.95, *p* < 0.001, *η*^2^ = 0.43]; participants purchased more snacks in the long-wait condition [*M* = 4.51, SD = 1.79] than in the short-wait condition [*M* = 1.93, SD = 1.47], a marginally significant effect of signal [*F*(1, 77) = 3.42, *p* = 0.07, *η*^2^ = 0.043]; participants purchased slightly more snacks in the signal-absent condition [*M* = 3.24, SD = 2.39] than in the signal-present condition [*M* = 2.88, SD = 1.72], and, of most importance, an interactive effect of wait duration and signal [*F*(1, 77) = 3.53, *p* = 0.06, *η*^2^ = 0.044; see Figure 4]. As we found in the previous study, when participants were told that their wait was dependent on other participants’ preferences, they purchased more products following a long wait (*M* = 3.70, SD = 1.56) than following a short wait [*M* = 2.57, SD = 1.85; *F*(1,77) = 4.74, *p* = 0.03]. However, when participants were told their wait time was dependent on unrelated system load, the wait did not affect their purchases [*M* = 2.73, SD = 1.79 vs. *M* = 3.04, SD = 1.61, respectively; *F*(1,77) = 0.30, *p* = 0.58].

**Table 5:** Summary of analyses of the effects of wait duration and signal about others’ behavior on the number of number of snacks purchased, perceived wait length, and wait annoyance in Study 3b.

Variable	Number of snacks	Log-transformed number of snacks	Perceived wait length	Wait annoyance
Wait duration	57.95^***^	1.21	165.82^***^	57.95^**^
Signal about others’ behavior	3.42^+^	0.17	0.20	3.42^+^
Wait duration × signal about others’ behavior	3.53^+^	4.18^*^	1.25	4.92^*^

Entries in the table represent *F*-statistics.

+ *p* < 0.1,

* *p* < 0.05,

** *p* < 0.01, and

*** *p* < 0.001.

A KS test for normality showed that the distribution of the number of snacks participants purchased deviated significantly from normal (*D* = 0.17, *p* < 0.001, skewness = −0.09, SE = 0.27). We therefore also analyzed the log transformation of this measure. A 2 (wait duration: short vs. long) × 2 (signal about others’ behavior: present vs. absent) ANOVA on the log-transformed number of snacks purchased revealed only an interactive effect of wait duration and signal [*F*(1, 77) = 4.18, *p* = 0.04, *η*^2^ = 0.051; all other *p*s > 0.2].

As expected, participants rated the short wait as shorter than the long wait (*M*_short_ = 2.26, SD_short_ = 1.02 vs. *M*_long_ = 5.11, SD_long_ = 0.93, *F*(1,77) = 165.82, *p* < 0.001, *η*^2^ = 0.68; all other *ps* > 0.2), indicating that participants were aware of their wait times. Participants’ reported annoyance was, once again, significantly affected by wait duration [*F*(1,77) = 57.95, *p* < 0.001, *η*^2^ = 0.43]. Participants perceived a longer wait to be more annoying than a shorter wait (*M*_long_ = 4.51, SD = 1.79 vs. *M*_short_ *=* 1.93, SD = 1.47). Signal had a marginally significant effect on wait annoyance [*F*(1,77) = 3.42, *p* = 0.07, *η*^2^ = 0.043]: participants perceived their wait as slightly more annoying in the no-signal condition than when a signal was present (*M*_no-signal_ *=* 3.24, SD_no-signal_ = 2.39 vs. *M*_signal_ = 2.88, SD_signal_ = 1.72). The effect of the interaction between wait duration and signal on wait annoyance was significant [*F*(1,77) = 4.92, *p* = 0.03, *η*^2^ = 0.06]. However, participants perceived the long wait as more annoying than the short wait both in the absence [*M*_long_ = 5.33, SD_long_ = 1.76 vs. *M*_short_ = 1.87, SD_short_ = 1.63, *F*(1,77) = 44.67, *p* < 0.001] and in the presence of a signal for others’ preferences [*M*_long_ = 3.90, SD_long_ = 1.59 vs. *M*_short_ = 2.00, SD_short_ = 1.31, *F*(1,77) = 15.84, *p* < 0.001].

##### Discussion

The results of Study 3b provide additional support for the notion that customers may use waiting time as an indication of others’ preferences, leading them to buy more after a longer wait. Once again, in a realistic setting, we found that longer waits, compared to shorter waits, led participants to purchase more snacks especially when participants believed their wait duration was affected by other participants’ preferences. Together, Study 3a and Study 3b reveal the role of social inference-making in the effect of wait duration on subsequent purchases and consumption.

## General discussion

Across four studies, we investigated how and why the duration of a wait affects subsequent purchase and consumption decisions. In an observation of café patrons, we found that the longer customers had to wait in line, the more money they spent on items purchased at the cashier stand after waiting (Study 1). We replicated this finding in a controlled lab experiment, where longer waits increased the number of items (stand-up comedy clips) selected for consumption despite inducing more negative emotional reactions than shorter waits (Study 2). Consistent with a social inference account, we further found that this effect of wait duration on the number of items selected for consumption (stand-up comedy clips; Study 3a) and for purchasing (snacks; Study 3b) occurred when the length of a wait could be assumed to depend on other customers’ preferences and was therefore likely to be perceived as indicative of value in the waited-for good or service. Furthermore, increased purchasing and consumption following long (vs. short) waits occurred despite greater annoyance from the long waits (studies 2 through 3b).

These results provide evidence that longer waits can increase subsequent purchasing and consumption of hedonic products even though they elicit more negative emotional reactions. The results also illustrate the key role played in that effect by social inferences; as noted, the effect emerges only when wait times are perceived to be dependent on other customers’ preferences (in the presence of socially based signals of value). When this condition applies (as indeed happens in most common service settings), a long wait may lead to the inference that the good or service being purchased is worth waiting for, which increases subsequent purchase and consumption.

### Theoretical implications

Despite the ubiquity of customer waiting experiences and the established behavioral literature on waiting, little research has examined how waiting might influence subsequent purchasing behavior (as opposed to, e.g., intentions to revisit the store or to spread word-of-mouth; Hui et al., 1997; Gorn et al., 2004; Giebelhausen et al., 2011; Craig et al., 2017). In an exception to this general rule, Koo and Fishbach (2010) showed that longer queues behind a customer increase the amount of money spent upon reaching the front of the line. The current research extends this literature by showing that prolonged waits, too, despite generating more negative emotional reactions than shorter waits, lead to increased purchasing when social inferences are made about waiting time. In so doing, this research helps elucidate the effects of waiting on customer reactions and, in particular, on actual purchasing behaviors, and sheds light on a psychological mechanism that underlies these effects.

The current research also takes a step toward disentangling the previously reported effects of waiting time on customers. Earlier studies on waiting have assumed that negative reactions to longer waits inevitably carry negative implications for firms (Taylor, 1994; Carmon et al., 1995; Hui and Tse, 1996; Antonides et al., 2002), but empirical findings have been mixed. More recent studies have shown some positive effects of extended waits. For example, longer waits can reduce stress among people waiting for an aversive event (Miller et al., 2008) and increase customers’ patience and the value they place on certain outcomes (Dai and Fishbach, 2013). The current study offers new insights into the multifaceted influence of time on waiting experiences. By showing that longer waits may boost purchases even when customers regard the wait as annoying, the current research undermines the typical assumption that when waiting is costly for customers, it can only damage firms’ bottom lines.

Our research also contributes to the development of behavioral theory by revealing direct and indirect relationships between waiting and other common customer behaviors. While a profound understanding of the complicated experience of waiting requires insights into the mechanisms that motivate customers and direct their behavior, these psychological mechanisms are rarely studied (for exceptions, see Hui and Tse, 1996; Munichor and Rafaeli, 2007; Koo and Fishbach, 2010; Kazinka et al., 2021). Most often, investigations into the impact of waiting are limited to more generic responses, such as affect and satisfaction. By showing the effect of social inferences on the relationship between waiting time and purchase behavior, our research may expose links between reactions to waiting and customer behaviors in other contexts.

### Practical implications

The idea that people may buy more hedonic products following a longer wait, as this research suggests, ostensibly contradicts previous findings and intuition on the negative reactions elicited by long waits. In particular, intuition suggests that customers who are annoyed by a long wait would prefer to “punish” the firm by taking their business elsewhere. Thus, it has been assumed that firms only suffer when customers must wait for a good or service, particularly when the wait is a long one. The present research suggests that when wait duration promotes inferences about the value of the item being waited for, longer waits can have positive implications for firms despite the fact that they generate more negative emotional reactions. Our analysis focused on *immediate* in-trip purchasing behavior of *non-abandoning* customers. However, coupled with proper management of abandonment rates and long-term consequences, longer waits may have a positive impact on subsequent purchases and consumption.

Our findings are especially relevant given the contemporary experience of waiting, in which a growing number of lines are virtual. With virtual waiting, customers operate in their own physical environment, isolating them from service agents and from other customers. In such service environments, customers cannot use queue length as a source of information, and the length of time spent waiting may play an important role in the process by which customers decide how much to buy and consume. Our research suggests that even in the absence of human interactions, customers can be encouraged to see longer waits as a sign of value in the waited-for good or service, for example, by making an explicit link between waiting time and item popularity (e.g., “please wait while other customers are being processed”).

### Limitations and future research

Our results suggest that longer waits may lead customers to buy more. However, in our lab experiments, participants were not explicitly informed about the option to stop their wait (they were informed that they could stop their participation at any time), whereas longer waits may also make customers more likely to abandon the line (e.g., Zohar et al., 2002; Xu et al., 2021). Because longer waits can have both positive and negative effects, it seems important that future research examines the overall effect of waiting on firms’ bottom lines. Such research could, for example, focus on whether increased purchases from customers who wait longer in line might compensate for lost sales due to customers who abandon the same line. Future research could also investigate the long-term consequences of waiting on customer loyalty, repeated store visits, and word-of-mouth. It might also be interesting to examine if there exists an “optimal waiting time” that balances all these gains and losses.

Another avenue for future research may concern the individual characteristics of the waiting customers. Three of the studies in the current research were conducted as lab experiments in order to control for factors that could potentially confound their results (such as distracting factors, different consumption reasons, etc.). Alongside the advantages of a lab experiment, this type of research has shortcomings that stem, for example, from the homogeneity of the participants or the use of an artificial setting (Peterson, 2001; Levitt and List, 2007). In particular, participants in our lab experiments were all students. Thus, although our controlled studies robustly established our hypotheses in cases in which customers are relatively young and educated, the setting of these studies also narrowed *a priori* the generalizability of their conclusions to this type of customers. Furthermore, the lab setting may have affected participants’ sense of time pressure, for example, by causing them to perceive less urgency to complete tasks than in real life. Our field study, which was conducted in a real consumption environment—a café—and therefore naturally included diverse types of customers with varying levels of time pressure, suggests that our findings may be generalizable across customers with different characteristics. Nevertheless, it would be interesting to examine whether responses to waits of different lengths vary across different types of customers and different levels of time pressure.

The current research also leaves open the question of when customers rely more versus less on social inferences from their wait. For example, we did not examine how product type changes our effects. Rather, the current research focused on responses to waiting for hedonic products (café items, stand-up comedy clips, and snacks), which impedes the generalizability of its findings to the context of waiting for utilitarian products. This might be particularly important considering that prior research suggests that social inferences may be less dominant when utilitarian (vs. hedonic) products are to be purchased (Poor et al., 2013; Schulze et al., 2014; Baek and Choo, 2015). Moreover, customers’ knowledge about the retail environment and purchased products may also affect the weight given to social inferences, as customers tend to draw inferences from others’ behavior particularly when they feel they have incomplete information (Deutsch and Gerard, 1955; Burnkrant and Cousineau, 1975). The exact role of these and other factors in determining when and to what extent customers use social signals needs to be tested.

Another interesting area of future research concerns other mechanisms that may contribute to increased purchases following longer waits. We focused on social inferences about the value of the waited-for items, showing that customers buy more following longer waits when wait times are taken as indicative of others’ preferences. However, as mentioned at the start of this paper, other underlying mechanisms could also contribute to the effect of wait duration. One such mechanism is self-control depletion (Muraven and Baumeister, 2000; Baumeister, 2002; Baumeister et al., 2008; Vohs et al., 2008). Long waits impose higher demands on customers than short waits and therefore require more continued self-regulation, which can be expected to deplete customer resources to a greater extent. Thus, after a long wait, customers’ attempts to exert self-control are less likely to succeed (Muraven and Baumeister, 2000; Baumeister, 2002), and they may find it harder to resist the temptation to buy (Vohs and Faber, 2007). Customers’ increased investment in longer waits may also encourage them to buy more in order to compensate for the perceived (sunk) cost of their waiting (Arkes and Blumer, 1985; Thaler, 1985; Ülkü et al., 2020; Kazinka et al., 2021). Emotional investment in waiting, and the consequent negative feelings (studies 2 through 3b; Osuna, 1985; Dubé et al., 1991; Zhou and Soman, 2003), may further encourage customers to buy more in order to improve their emotional state (Arnold and Reynolds, 2009). Although self-control depletion, sunk costs, and mood repair cannot easily account for the interactions that emerged in studies 3a and 3b, these mechanisms may at times account for increased purchases following longer waits. We would welcome future research to identify the specific conditions under which each of these mechanisms comes into play.

Another open question pertains to the length of the wait. Our findings seem to suggest that the effect of wait duration is robust when the wait is no more than a few minutes. However, whether and how different wait lengths change the effect remains to be investigated. For example, a very long wait may indicate a service failure (Mandelbaum and Shimkin, 2000; Janakiraman et al., 2011), rather than higher value in whatever is being waited for. Whether an absolute length threshold exists or whether the threshold is goal-and context-dependent remains to be investigated.

Overall, this research suggests that scholars, customers, and practitioners alike would benefit from better understanding of the multifaceted effects of waiting on customer behavior and the role played by social inferences in those effects.

## Data availability statement

Data and study materials are available at: https://doi.org/10.6084/m9.figshare.c.6185737.v1.

## Ethics statement

The studies involving human participants were reviewed and approved by University of Florida. The patients/participants provided their written informed consent to participate in this study. Written informed consent was obtained from the individual(s) for the publication of any identifiable images or data included in this article.

## Author contributions

NM collected and analyzed the data of the field study and wrote the first draft of the manuscript. NM and AC designed the lab experiments, collected their data, analyzed those data and wrote sections of the manuscript, read, and approved the submitted version. All authors contributed to the article and approved the submitted version.

## Conflict of interest

The authors declare that the research was conducted in the absence of any commercial or financial relationships that could be construed as a potential conflict of interest.

## Publisher’s note

All claims expressed in this article are solely those of the authors and do not necessarily represent those of their affiliated organizations, or those of the publisher, the editors and the reviewers. Any product that may be evaluated in this article, or claim that may be made by its manufacturer, is not guaranteed or endorsed by the publisher.
